# Factores determinantes en el retraso de la solicitud de asistencia sanitaria en mujeres con síndrome coronario agudo

**DOI:** 10.1016/j.aprim.2026.103618

**Published:** 2026-09-22

**Authors:** Marta Parellada-Vendrell, Sílvia Pérez-Ortega, Nuria Romeu-Mirabete, Montserrat Venturas, Rut Andrea, Adelaida Zabalegui

**Affiliations:** a Unidad de Cuidados Cardiacos Agudos, Instituto Clínic Cardiovascular, Hospital Clínic de Barcelona, Barcelona, España; b Universidad de Barcelona, Barcelona, España; c Instituto de Investigaciones Biomédicas August Pi i Sunyer (IDIBAPS), Barcelona, España; d Instituto Clínic Cardiovascular, Hospital Clínic de Barcelona, Barcelona, España; e Subdirección de Enfermería, Investigación y Docencia, Hospital Clínic de Barcelona, Barcelona, España

**Keywords:** Síndrome coronario agudo, Demora prehospitalaria, Salud de la mujer, Toma de decisiones, Primera asistencia médica, Rol de género, Acute coronary syndrome, Pre-hospital delay, Women's health, Decision making, First medical responders, Gender role

## Abstract

**Objetivo:**

Identificar los factores asociados al retraso en la solicitud de asistencia sanitaria en mujeres con síndrome coronario agudo (SCA).

**Diseño:**

Estudio observacional, transversal y unicéntrico.

**Emplazamiento:**

Ámbito hospitalario (unidad de cuidados cardiacos agudos de un hospital de tercer nivel).

**Participantes:**

Selección consecutiva de mujeres ingresadas y supervivientes a un SCA durante un año; finalmente se incluyeron 80 participantes.

**Mediciones principales:**

Variables sociodemográficas, de riesgo cardiovascular, clínicas, contextuales, cognitivas, de conocimiento, conductuales y sobre roles de género. Se identificaron los factores asociados a un tiempo de decisión (TD) > 120 minutos mediante regresión de Poisson con varianza robusta, calculando las razones de prevalencia ajustadas (RPa) e intervalos de confianza del 95% (IC 95%), ajustado por edad.

**Resultados:**

La mediana de TD fue de 120 minutos (RIC: 54-583). La ausencia de percepción de gravedad fue el principal factor asociado a la demora (RPa = 3,62; IC 95%: 1,80-7,29; p < 0,001). Residir fuera del núcleo urbano también se vinculó con un mayor TD (RPa = 1,51; IC 95%: 1,06-2,15; p = 0,021). El antecedente de síndrome ansioso-depresivo mostró una tendencia marginal hacia el retraso (RPa = 1,39; IC 95%: 0,99-1,95; p = 0,053), mientras que presentar mareo-inestabilidad se relacionó con una menor prevalencia de demora (RPa = 0,31; IC 95%: 0,10-0,97; p = 0,044).

**Conclusiones:**

El retraso en la solicitud de asistencia sanitaria en mujeres con SCA se asocia con factores sociodemográficos, clínicos y, fundamentalmente, cognitivos. Es prioritario integrar estos hallazgos en el diseño de estrategias e intervenciones comunitarias para reducir los tiempos de respuesta y mejorar la salud cardiovascular de la mujer.

## Abstract (en)

**Objective:**

To identify the factors associated with delays in seeking healthcare among women with Acute Coronary Syndrome (ACS).

**Design:**

Observational, cross-sectional and single-center study.

**Setting:**

Hospital setting (Acute Cardiac Care Unit of a tertiary hospital).

**Participants:**

Consecutive selection of women admitted for and surviving an ACS over a one-year period; 80 participants were ultimately included.

**Main measurements:**

Sociodemographic, cardiovascular risk, clinical, contextual, cognitive, knowledge, behavioural, and gender role variables were analysed. Factors associated with a decision time (DT) > 120 minutes were identified using Poisson regression with robust variance, calculating adjusted prevalence ratios (aPR) and 95% confidence intervals (95% CI), adjusted for age.

**Results:**

The median DT was 120 minutes (IQR: 54-583). The lack of perceived severity was the main factor associated with delay (aPR = 3.62; 95% CI: 1.80–7.29; p < 0.001). Residing outside the urban area was also associated with a longer DT (aPR = 1.51; 95% CI: 1.06–2.15; p = 0.021). A history of anxiety-depressive syndrome showed a borderline trend toward delay (aPR = 1.39; 95% CI: 0.99–1.95; p = 0.053), whereas presenting with dizziness/instability was associated with a lower prevalence of delay (aPR = 0.31; 95% CI: 0.10–0.97; p = 0.044).

**Conclusions:**

Delays in seeking healthcare among women with ACS are associated with sociodemographic, clinical, and, fundamentally, cognitive factors. Integrating these findings into the design of community-based strategies and interventions is a priority to reduce response times and improve women's cardiovascular health.

## Introducción

La enfermedad cardiovascular causa el 35% de las muertes anuales en mujeres, siendo el síndrome coronario agudo (SCA) la primera causa de mortalidad^1^. La detección temprana y el control de los factores de riesgo cardiovascular (FRCV) son fundamentales para mejorar la salud cardiovascular y reducir la morbimortalidad^1^, ^2^.

El retraso en la demanda de atención sanitaria se define como el intervalo de tiempo entre el inicio de los síntomas y la solicitud de atención médica. Diversas publicaciones identifican a las mujeres como un grupo de riesgo, ya que el intervalo de tiempo hasta la toma de decisión es significativamente más prolongado que en los hombres, lo que favorece presentaciones clínicas evolucionadas e impacta negativamente en el pronóstico^2^, ^3^, ^4^, ^5^.

Pese a las estrategias para garantizar el acceso temprano y equitativo al tratamiento de reperfusión coronaria tras el primer contacto médico^6^, en el entorno prehospitalario persisten lagunas sobre los determinantes específicos que influyen en el tiempo de decisión (TD) y motivan retrasos en la demanda de atención en mujeres. Analizar estos factores desde un enfoque multidimensional que integre la perspectiva de género^7^, ^8^, ^9^ permitiría diseñar estrategias dirigidas e implantar intervenciones comunitarias para reducir este lapso de tiempo y mejorar los resultados en salud. El objetivo del estudio es identificar los factores asociados al retraso en la solicitud de asistencia sanitaria en mujeres con SCA.

## Métodos

### Diseño del estudio y participantes

Estudio observacional, transversal y unicéntrico con inclusión consecutiva de mujeres ingresadas y supervivientes a un SCA en la unidad de cuidados cardiacos agudos (UCCA) de un hospital terciario entre enero de 2022 y enero de 2023. Se excluyeron pacientes con deterioro cognitivo, síndrome confusional, barrera idiomática, revascularización quirúrgica emergente, fallecimiento previo o rechazo a participar.

### Recogida de datos y variables

Los datos se obtuvieron mediante revisión de la historia clínica y entrevista estructurada con la participante, realizada a partir de las 12 horas de ingreso en la UCCA, utilizando un cuestionario *ad hoc,* cuya validez de contenido fue consensuada por expertos clínicos coronarios y pilotada para asegurar su comprensibilidad y aplicabilidad. La entrevista (45-60 minutos) se realizó siempre que la paciente se encontrara consciente, orientada y hemodinámicamente estable. Aquellos casos que no cumplían estos criterios (inestabilidad hemodinámica, dispositivo de asistencia mecánica circulatoria, intubación endotraqueal o sedación) fueron reevaluados diariamente hasta permitir la comunicación verbal; en caso contrario, se consideraron pérdidas.

La variable dependiente fue el TD, definido como el intervalo (minutos) transcurrido desde el inicio de la sintomatología hasta la solicitud de asistencia sanitaria. Como variables independientes se incluyeron factores sociodemográficos, FRCV, clínicos, contextuales, cognitivos, de conocimiento, conductuales y roles de género. Al realizarse la entrevista postingreso, la reconstrucción del TD y las variables cognitivas, conductuales y contextuales tienen carácter retrospectivo, estando expuestas a sesgo de recuerdo y reinterpretación tras conocer el diagnóstico. Para minimizarlo, los datos horarios reportados se contrastaron con los registros del servicio de emergencias médicas (SEM) y centros sanitarios en los que fueron atendidas.

Las variables de género se evaluaron con el índice de sobrecarga de género (ISG), instrumento exploratorio *ad hoc* de carácter formativo^10^ basado en la literatura sobre determinantes sociales de la salud^1^, ^11^, ^12^. Consta de 4 indicadores: 1) rol de cuidadora principal; 2) cargas familiares; 3) priorización del bienestar familiar sobre el propio; y 4) preocupación por la alteración de la dinámica familiar tras el ingreso. Cada ítem se evaluó de forma dicotómica, asignando un punto por cada respuesta afirmativa, categorizándolo en carga baja-media (0-2 puntos) y alta (3-4 puntos).

### Análisis estadístico

Las variables categóricas se expresaron en frecuencias y porcentajes. La normalidad de las variables cuantitativas se analizó mediante la prueba de Kolmogórov-Smirnov, describiéndose como media y desviación estándar (DE) en distribución normal, y mediana y rango intercuartílico (RIC) en no normal. Puesto que la distribución del TD fue asimétrica y no existe un umbral clínico estandarizado, se empleó la mediana (> 120 minutos vs. ≤ 120 minutos) como punto de corte epidemiológico. Al ser los datos ausentes < 5% y aleatorios se analizaron los casos disponibles.

La asociación entre variables categóricas se evaluó con la prueba Chi cuadrado o el test de Fisher (cuando más del 20% de las frecuencias esperadas eran < 5). Para variables cuantitativas se utilizó la «t» de Student en distribución normal, o U de Mann-Whitney en no normal.

Para identificar los factores asociados al retraso, se calcularon las razones de prevalencia ajustadas (RPa) e intervalos de confianza del 95% (IC 95%) mediante regresión de Poisson con varianza robusta^13^. Para prevenir el sobreajuste, la selección de covariables se realizó bajo el principio de parsimonia, limitándose a 5 variables con relevancia clínica o significación en el análisis univariante (p < 0,10): edad, residir fuera del núcleo urbano, antecedentes de síndrome ansioso-depresivo, presencia de mareo-inestabilidad y ausencia de percepción de gravedad durante el evento. La relación fue 8 eventos por variable, asegurando la estabilidad de las estimaciones.

La ausencia de multicolinealidad se verificó con el factor de inflación de la varianza (VIF), y el ajuste del modelo mediante la prueba ómnibus y la bondad de ajuste de Pearson. De forma exploratoria, la capacidad de clasificación se evaluó con el área bajo la curva ROC (AUC), sensibilidad y especificidad. Se consideró significativo un valor de p < 0,05, usando IBM SPSS v.25 y siguiendo la declaración STROBE^14^ y el decálogo PROGENEROS^15^.

### Responsabilidades éticas

El estudio fue aprobado por el Comité de Ética de la Investigación con Medicamentos (HCB/2021/1221) conforme la Ley de Investigación Biomédica 14/2007 y la Declaración de Helsinki^16^. La información clínica se trató de forma confidencial y anonimizada y se obtuvo el consentimiento informado de las participantes.

## Resultados

Durante el período de estudio 111 mujeres ingresaron en la UCCA con orientación diagnóstica de SCA. Tras excluir 31 casos se incluyeron 80 pacientes (fig. 1).

**Figura 1:**
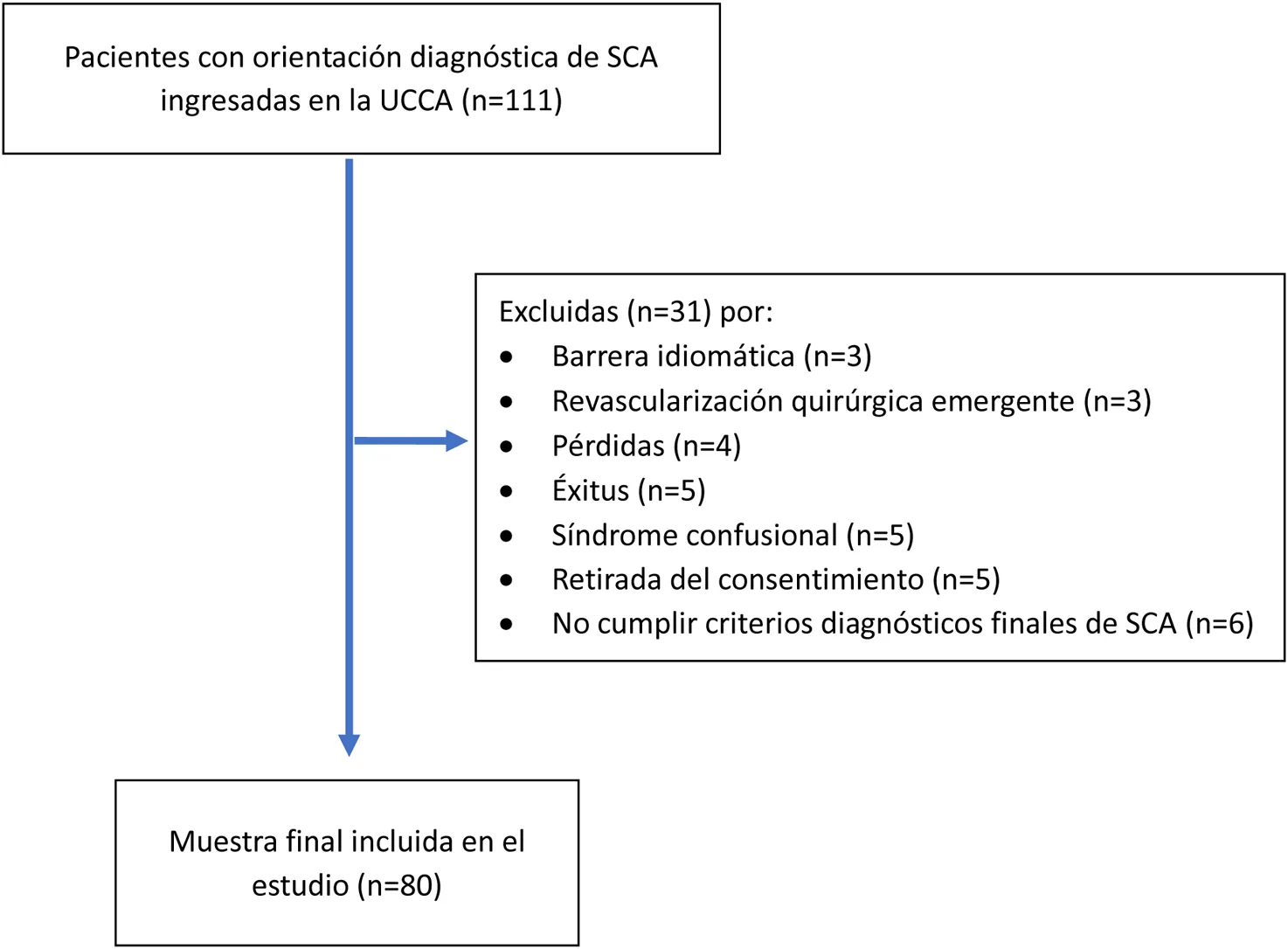
Esquema del estudio. SCA: síndrome coronario agudo; UCCA: unidad de cuidados cardiacos agudos.

### Características sociodemográficas y clínicas

La edad media fue 68 ± 13 años (51,2% eran ≥ 70 años), un 27,5% vivían solas, un 23,8% eran viudas y el 75% residía en el núcleo urbano de Barcelona ciudad. En el aspecto clínico, el 52,5% presentaba 5 o más FRCV, el 66,3% fue diagnosticada de infarto agudo de miocardio con elevación del segmento ST y un 8,8% ingresó en clase funcional Killip-Kimball III-IV^17^.

A todas las participantes se les realizó coronariografía (68,8% urgente, 26,2% preferente y diferida 5%), objetivándose enfermedad coronaria obstructiva en un 81,3%. El resto se diagnosticó de infarto agudo de miocardio sin lesiones coronarias obstructivas (17,5%) e isquemia sin enfermedad coronaria obstructiva (1,2%). Se hizo revascularización en el 80% de los casos, percutánea (72,5%) y quirúrgica (7,5%).

### Tiempo de decisión

La mediana del TD fue 120 minutos (RIC:54-583). El análisis bivariante (Tabla 1, Tabla 2, Tabla 3, Tabla 4) asoció la consulta precoz (TD ≤ 120 minutos) con residir en el núcleo urbano (87,5% vs. 62,5%; p = 0,010) y presentar ≥ 4 síntomas (27,5% vs. 10%; p = 0,044). Respecto a la salud mental el 35% presentaba síndrome ansioso-depresivo, observándose una tendencia hacia mayor TD (45% vs. 25%; p = 0,061). Asimismo, la conducta inicial ante la sintomatología difirió entre grupos (p < 0,001), destacando que el 35% de las mujeres con retraso prolongado mantuvo sus actividades previstas. El recurso asistencial utilizado como primer contacto médico mostró diferencias significativas (p = 0,001), mientras que en el grupo con TD precoz (≤ 120 minutos) predominó la activación del SEM (75% vs. 37,5%), en el de retraso (> 120 minutos) optaron por otros recursos asistenciales (62,5% vs. 25%), como acudir a urgencias (25%) o consultar con atención primaria (37,5%). Finalmente, el 61,3% reconoció haber demorado la solicitud de ayuda (p < 0,001), por no reconocer la gravedad del episodio (73,5%) o evitar preocupar a la familia (18,4%).

**Tabla 1:** Características sociodemográficas y factores de riesgo cardiovascular en mujeres con síndrome coronario agudo según el tiempo de decisión

Variables	Total (n = 80)	≤ 120 min (n = 40)	> 120 min (n = 40)	p-value
**Factores sociodemográficos**				
Edad (años) [MEDIA ± DE]	68 ± 13	68 ± 14	68 ± 12	0,987
Edad ≥ 70 años	41 (51,2)	21 (52,5)	20 (50,0)	0,823
Vivir sola	22 (27,5)	10 (25,0)	12 (30,0)	0,617
Residencia fuera del núcleo urbano	20 (25,0)	5 (12,5)	15 (37,5)	**0,010**
*Estado civil*				0,654
Casada-unión libre	38 (47,5)	20 (50,0)	18 (45,0)	
Soltera-separada/divorciada-viuda	42 (52,5)	20 (50,0)	22 (55,0)	
*Ocupación*				0,251
Activa laboralmente-ama de casa	31 (38,8)	18 (45,0)	13 (32,5)	
Pensionista	49 (61,3)	22 (55,0)	27 (67,5)	
**Factores de riesgo cardiovascular**				
N.° FRCV (MEDIANA [RIC])	5 (3-6)	4 (3-5)	5 (4-7)	0,067
≥ 5 FRCV	42 (52,5)	18 (45,0)	24 (60,0)	0,179
Postmenopausia^f^	69 (87,3)	33 (84,6)	36 (90,0)	0,518
Dislipidemia	51 (63,7)	22 (55,0)	29 (72,5)	0,104
Hipertensión arterial	49 (61,3)	23 (57,5)	26 (65,0)	0,491
Cardiopatía isquémica familiar	33 (41,3)	15 (37,5)	18 (45,0)	0,496
Sobrepeso	30 (37,5)	16 (40,0)	14 (35,0)	0,644
Estrés percibido	29 (36,3)	11 (27,5)	18 (45,0)	0,104
Síndrome ansioso depresivo	28 (35,0)	10 (25,0)	18 (45,0)	0,061
Diabetes mellitus	25 (31,3)	10 (25,0)	15 (37,5)	0,228
Tabaquismo activo	24 (30,0)	11 (27,5)	13 (32,5)	0,626
Obesidad	23 (28,7)	12 (30,0)	11 (27,5)	0,805
Insuficiencia renal crónica	15 (18,8)	8 (20,0)	7 (17,5)	0,775
Cardiopatía isquémica previa	13 (16,3)	6 (15,0)	7 (17,5)	0,762

Los datos se expresan en n (%), media (DE) o mediana (RIC).

DE: desviación estándar; FRCV: factores de riesgo cardiovascular; RIC: rango intercuartílico.

f Calculado sobre n = 79 casos válidos registrados. Los datos en negrita representan resultados estadísticamente significativos (p < 0,05).

**Tabla 2:** Factores clínicos y diagnósticos en mujeres con síndrome coronario agudo según el tiempo de decisión

Variables	Total (n = 80)	≤ 120 min (n = 40)	> 120 min (n = 40)	p-value
**Factores clínicos y diagnósticos**				
Dolor torácico	77 (96,3)	39 (97,5)	38 (95,0)	1,000
Intensidad Dolor EVN ≥ 8^g^	54 (74,0)	27 (73,0)	27 (75,0)	0,844
Localización centrotorácica^h^	56 (73,7)	28 (71,8)	28 (75,7)	0,701
Localización epigástrica	5 (6,6)	1 (2,6)	4 (10,8)	0,194
Características opresivas	62 (82,7)	34 (87,2)	28 (77,8)	0,283
Irradiación^i^	57 (74,0)	30 (76,9)	27 (71,1)	0,557
Clase funcional Killip III-IV	7 (8,8)	2 (5,0)	5 (12,5)	0,432
≥ 4 síntomas	15 (18,8)	11 (27,5)	4 (10,0)	**0,044**
Diaforesis	39 (48,8)	22 (55,0)	17 (42,5)	0,263
Náuseas	28 (35,0)	14 (35,0)	14 (35,0)	1,000
Disnea	25 (31,3)	11 (27,5)	14 (35,0)	0,469
Debilidad	19 (23,8)	14 (35,0)	5 (12,5)	**0,018**
Fatiga	17 (21,3)	8 (20,0)	9 (22,5)	0,785
Vómitos	15 (18,8)	10 (25,0)	5 (12,5)	0,152
Mareo-inestabilidad	11 (13,8)	9 (22,5)	2 (5,0)	**0,023**
IAM subagudo	4 (5,0)	0 (0,0)	4 (10,0)	0,116
FEVI reducida (≤ 40%)^j^	15 (19,0)	7 (17,9)	8 (20,0)	0,816
*Tipo de SCA*				0,813
IAMSEST-angina inestable	27 (33,8)	13 (32,5)	14 (35,0)	
IAMCEST	53 (66,3)	27 (67,5)	26 (65,0)	
Localización del IAMCEST^k^				
Inferior	17 (32,1)	9 (33,3)	8 (30,8)	
Anterior	16 (30,2)	9 (33,3)	7 (26,9)	
Más de una localización	14 (26,4)	7 (25,9)	7 (26,9)	
Otras localizaciones	6 (11,3)	2 (7,4)	4 (15,4)	
*Encuentros angiográficos*				0,152
EAC obstructiva	65 (81,3)	30 (75,0)	35 (87,5)	
MINOCA-INOCA	15 (18,8)	10 (25,0)	5 (12,5)	
*Complicaciones del SCA*				0,152
No	54 (67,5)	24 (60,0)	30 (75,0)	
Sí	26 (32,5)	16 (40,0)	10 (25,0)	
Tipo de complicaciones^l^				
PCR	5 (19,2)	4 (25,0)	1 (10,0)	
Arritmias	21 (80,8)	13 (81,3)	8 (80,0)	
Aneurisma ventricular	2 (7,7)	0 (0,0)	2 (20,0)	
Complicación mecánica aguda	1 (3,8)	0 (0,0)	1 (10,0)	

Los datos se expresan en n (%).

EVN: Escala verbal numérica; FEVI: Fracción de eyección del ventrículo izquierdo; SCA: Síndrome Coronario Agudo; IAMSEST: Infarto agudo de miocardio sin elevación del ST; IAMCEST: Infarto agudo de miocardio con elevación del ST; EAC: Enfermedad arterial coronaria; MINOCA: infarto agudo de miocardio con coronarias sin lesiones obstructivas; INOCA: Isquemia con enfermedad coronaria no obstructiva, PCR: Parada cardiorrespiratoria.

g Calculado sobre n = 73 casos válidos registrados.

h Calculado sobre n = 76 casos válidos registrados.

i Calculado sobre n = 77 casos válidos registrados.

j Calculado sobre n = 79 casos válidos registrados.

k Porcentajes sobre el subgrupo IAMCEST (n = 53).

l Porcentajes sobre el subgrupo con complicaciones (n = 26). Los datos en negrita representan resultados estadísticamente significativos (p < 0,05).

**Tabla 3:** Factores contextuales, cognitivos y de conocimiento en mujeres con síndrome coronario agudo según el tiempo de decisión

Variables	TOTAL (n = 80)	≤ 120 min (n = 40)	> 120 min (n = 40)	p-value
**Factores contextuales durante los síntomas**				
Ausencia de acompañantes	24 (30,0)	11 (27,5)	13 (32,5)	0,626
Domicilio habitual	59 (73,8)	30 (75,0)	29 (72,5)	0,799
Aparición en reposo^m^	28 (36,8)	13 (34,2)	15 (39,5)	0,634
Inicio nocturno de los síntomas	27 (33,8)	14 (35,0)	13 (32,5)	0,813
Inicio en fin de semana o festivo	16 (20,0)	9 (22,5)	7 (17,5)	0,576
**Factores cognitivos**				
Ausencia de percepción de gravedad	48 (60,0)	14 (35,0)	34 (85,0)	**<** **0,001**
Atribución no cardiaca de los síntomas^n^	50 (64,1)	17 (43,6)	33 (84,6)	**<** **0,001**
Presencia de síntomas prodrómicos	25 (31,3)	13 (32,5)	12 (30,0)	0,809
**Factores de conocimiento**				
Desconocimiento del SCA	19 (23,8)	10 (25,0)	9 (22,5)	0,793
Falta de autorreconocimiento del SCA	59 (73,8)	25 (62,5)	34 (85,0)	**0,022**
Atribución del SCA como enfermedad masculina	14 (17,7)	7 (17,5)	7 (17,5)	0,958
Desconocimiento de los FRCV	18 (22,5)	8 (20,0)	10 (25,0)	0,592

Los datos se expresan en n (%).

FRCV: factores de riesgo cardiovascular; SCA: síndrome coronario agudo.

m Calculado sobre n = 76 casos válidos registrados.

n Calculado sobre n = 78 casos válidos registrados. Los datos en negrita representan resultados estadísticamente significativos (p < 0,05).

**Tabla 4:** Factores conductuales y roles de género en mujeres con síndrome coronario agudo según el tiempo de decisión

Variables	Total (n = 80)	≤ 120 min (n = 40)	> 120 min (n = 40)	Valor de p
**Factores conductuales**				
*Conducta inicial tras la aparición de los síntomas*				**<** **0,001**
Demanda inmediata de asistencia	14 (17,5)	14 (35,0)	0 (0,0)	
Mantenimiento actividades previstas	17 (21,3)	3 (7,5)	14 (35,0)	
Reposo a la espera de remisión	23 (28,7)	10 (25,0)	13 (32,5)	
Medidas farmacológicas y no farmacológicasñ	26 (32,5)	13 (32,5)	13 (32,5)	
*Motivo desencadenante de la consulta*				0,127
Exacerbación del cuadro clínico	36 (45,0)	14 (35,0)	22 (55,0)	
Persistencia de la sintomatología	23 (28,7)	15 (37,5)	8 (20,0)	
Recomendación de terceras personas	16 (20,0)	7 (17,5)	9 (22,5)	
Otros motivos	5 (6,3)	4 (10,0)	1 (2,5)	
*Decisión de solicitud de ayuda*				0,371
Acompañantes/testigos	40 (50,0)	22 (55,0)	18 (45,0)	
La propia paciente	40 (50,0)	18 (45,0)	22 (55,0)	
*Primer contacto médico*				**0,001**
SEM	45 (56,3)	30 (75,0)	15 (37,5)	
Otros	35 (43,8)	10 (25,0)	25 (62,5)	
Urgencias hospitalarias	15 (18,8)	5 (12,5)	10 (25,0)	
Atención primaria	20 (25,0)	5 (12,5)	15 (37,5)	
*Demora percibida*				**<** **0,001**
Considera consulta precoz	31 (38,8)	29 (72,5)	2 (5,0)	
Reconoce demora en la consulta	49 (61,3)	11 (27,5)	38 (95,0)	
*Motivos de demora*^o^				
Dificultad en reconocer la gravedad	36 (73,5)	7 (63,6)	29 (76,3)	
Evitar causar problemas a la familia	9 (18,4)	3 (27,3)	6 (15,8)	
Otros motivos	4 (8,2)	1 (9,1)	3 (7,9)	
**Roles de género**				
*Índice de sobrecarga de género*				0,108
Carga de género baja-media (0-2)	49 (61,3)	21 (52,5)	28 (70,0)	
Carga de género alta (3-4)	31 (38,8)	19 (47,5)	12 (30,0)	
ISG (mediana [RIC])	2 (1-3)	2 (2-3)	2 (1-3)	0,712

Los datos se expresan en n (%) o mediana (RIC).

ISG: índice de sobrecarga de género; RIC: rango intercuartil; SEM: servicio de emergencias médicas.

ñ Incluye el uso de analgésicos (n = 6), cafinitrina (n = 5), ansiolíticos (n = 4), antiácidos (n = 2), otros fármacos (n = 3) y medidas no farmacológicas como calor local o infusiones (n = 4).

o Porcentajes calculados sobre el subgrupo de pacientes que reconocieron demora en la consulta n = 49. Los datos en negrita representan resultados estadísticamente significativos (p < 0,05).

### Perspectiva de género

El ISG no mostró asociación estadística con el TD (p = 0,108), aunque el 61,3% de las mujeres con carga alta de género consultó precozmente frente al 42,9% con carga baja-media. El ISG se relacionó con la edad (p = 0,007), concentrándose la mayor carga de género en las mujeres < 70 años (67,7%).

Al desglosar el índice se observó que el rol de cuidadora principal (57,5% de la muestra) y las cargas familiares directas (23,8% del total) fueron significativamente mayores en las mujeres < 70 años frente a las ≥ 70 años (74,4% vs. 41,5%; p = 0,003 y 38,5% vs. 9,8%; p = 0,003). La priorización del bienestar familiar fue un aspecto transversal que afectó al 85% de las mujeres sin diferencias por edad (84,6% vs. 85,4%; p = 0,925), manteniéndose homogénea la preocupación por la alteración de la dinámica familiar tras el ingreso (52,5% del total; 56,4% vs. 48,8%; p = 0,495).

### Factores asociados a mayor tiempo de decisión

En el análisis univariante (tabla 5), la ausencia de percepción de gravedad (RP = 3,78; p < 0,001), la atribución no cardíaca de los síntomas (RP = 3,08; p = 0,003), residir fuera del núcleo urbano (RP = 1,80; p = 0,003) y el número de FRCV (RP = 1,13; p = 0,021) se asociaron con mayor TD. Asimismo, el síndrome ansioso-depresivo, la debilidad y la falta de autorreconocimiento del SCA mostraron una tendencia marginal (p < 0,10).

**Tabla 5:** Análisis univariante y multivariante de los factores asociados al retraso en el tiempo de decisión (> 120 minutos) en mujeres con SCA

Variables	Univariante		Multivariante	
	RP cruda (IC 95%)	Valor de p	RP ajustada* (IC 95%)	p-value
Residir fuera del núcleo urbano	1,80 (1,22-2,66)	**0,003**	1,51 (1,06-2,15)	**0,021**
Número de FRCV	1,13 (1,02-1,26)	**0,021**	-	
Presencia Síndrome ansioso-depresivo	1,52 (0,99-2,31)	0,051	1,39 (0,99-1,95)	0,053
Presentar ≥ 4 síntomas	0,48 (0,20-1,15)	0,099	-	
Presencia de mareo-inestabilidad	0,33 (0,09-1,18)	0,088	0,31 (0,10-0,97)	**0,044**
Presencia de debilidad	0,46 (0,21-1,00)	0,051	-	
Ausencia de percepción de gravedad	3,78 (1,80-7,95)	**<** **0,001**	3,62 (1,80-7,29)	**<** **0,001**
Atribución no cardiaca de los síntomas	3,08 (1,47-6,43)	**0,003**	-	
Falta de autorreconocimiento del SCA	2,02 (0,99-4,11)	0,053	-	
Índice de sobrecarga de género (alta)	0,68 (0,41-1,12)	0,131	-	
Edad (variable ajuste)	1,00 (0,98-1,02)	0,987	1,00 (0,99-1,02)	0,475

FRCV: factores de riesgo cardiovascular; IC: intervalo de confianza; RP: razón de prevalencia; SCA: síndrome coronario agudo.

(-): Variables no incluidas en el modelo multivariante final.

*RP ajustada por todas las variables incluidas en el modelo final (edad, residir fuera del núcleo urbano, presencia de síndrome ansioso-depresivo, presencia de mareo-inestabilidad y ausencia de percepción de gravedad durante el evento agudo).

Área bajo la curva  = 0,864 (IC 95%: 0,783-0,946; p < 0,001).

Capacidad de clasificación global del 82,5%; sensibilidad del 92,5% y especificidad del 72,5%.

Prueba ómnibus (p = 0,002).

Bondad de ajuste de Pearson (valor/gl = 0,468).

Ausencia de multicolinealidad (VIF < 1,1; rango: 1,01-1,08; índice de condición máximo = 17,1).

Los datos en negrita representan resultados estadísticamente significativos (p < 0,05).

En el análisis multivariado (tabla 5) la ausencia de percepción de gravedad se mantuvo como la variable con mayor fuerza de asociación, triplicando la prevalencia de un TD > 120 minutos (RPa = 3,62; IC 95%: 1,80-7,29; p < 0,001). Residir fuera del núcleo urbano se relacionó de forma independiente con una mayor proporción de consulta tardía (RPa = 1,51; IC 95%: 1,06-2,15; p = 0,021). En contraposición, la presencia de mareo-inestabilidad se asoció inversamente con el retraso, observándose una menor prevalencia de demora (RPa = 0,31; IC 95%: 0,10-0,97; p = 0,044). El síndrome ansioso-depresivo conservó una tendencia marginal hacia el retraso (RPa = 1,39; IC 95%: 0,99-1,95; p = 0,053).

El modelo mostró un ajuste global (prueba ómnibus, p = 0,002), bondad de ajuste de Pearson sin sobredispersión (*valor/gl* = 0,468) ni multicolinealidad (VIF < 1,1; rango: 1,01-1,08). Como análisis exploratorio, el AUC fue 0,864 (IC 95%: 0,783-0,946; p < 0,001), con una clasificación global del 82,5% (sensibilidad: 92,5% y especificidad: 72,5%).

## Discusión

En la ruta asistencial del SCA se describen diferentes intervalos de tiempo desde la aparición de los síntomas hasta la instauración del tratamiento. Su medición permite evaluar retrasos atribuibles al sistema o al paciente, identificando áreas de mejora mediante indicadores de calidad^6^. Este estudio analiza el TD, un intervalo influenciado por múltiples factores que depende de la perspectiva del paciente y sus acompañantes o testigos.

Según los resultados obtenidos, tanto la mediana del TD como el porcentaje de mujeres con demoras > 120 minutos son superiores a los descritos por Sederholm Lawesson et al^4^., quienes evidenciaron diferencias por sexo (90 minutos y 40% en mujeres vs. 60 minutos y 30% en hombres), y donde coincidimos con los autores en que reducir el retraso atribuible al paciente tiene un gran potencial de mejora^4^.

La ausencia de percepción de gravedad durante el evento es un factor clave en la toma de decisiones y en la búsqueda de atención sanitaria en las mujeres con SCA. Más de la mitad de la muestra no percibió la gravedad del episodio, hecho que implica mayor TD, retrasar el primer contacto médico, acceder a la red asistencial de forma tardía y demorar las opciones terapéuticas establecidas en las guías de práctica clínica^6^, ^18^, ^19^. Además, conlleva complicaciones inherentes a la enfermedad, tanto en la fase aguda como a largo plazo, con el riesgo de arritmias ventriculares o insuficiencia cardiaca secundarias a la necrosis y disfunción miocárdica^20^, ^21^.

La evidencia describe cómo ciertos síntomas (sudor frío en mujeres y miedo/angustia en hombres) son percibidos como amenazantes, favoreciendo la búsqueda de atención y asociándose con un menor retraso del paciente^4^. Según Arrebola-Moreno et al.^22^ atribuir los síntomas a causas cardíacas y la gravedad percibida aceleran la solicitud de ayuda. Mirzaei et al.^3^ señalan que la presentación abrupta de la sintomatología (vs. gradual) acorta el TD.

*A priori*, cabría esperar que la presencia de dolor torácico (según la definición de la *American Heart Association*)^23^, su elevada intensidad o una clase funcional Killip-Kimball^17^ avanzada motivarían una consulta precoz. Como muestra la tabla 2, la gran mayoría presentó dolor torácico, desmitificando la creencia de que las mujeres con SCA no presentan este síntoma. Sin embargo, su efecto sobre el TD no pudo evaluarse debido al reducido número de pacientes sin dicho síntoma. Esto sugiere una posible brecha de conocimiento sobre los síntomas isquémicos en la mujer^24^, ^25^, ^26^, ^27^, de ahí que casi dos terceras partes atribuyeran la sintomatología a causas no cardiacas y solo una cuarta parte consideró estar sufriendo un SCA. Por otro lado, presentar mareo-inestabilidad se asoció de forma independiente con menor retraso en la demanda de atención sanitaria. En cambio, residir fuera del núcleo urbano se vinculó significativamente con mayor TD, debido probablemente a que el entorno urbano ofrece mayor concentración de recursos y facilita el acceso a los servicios de salud, generando situaciones de inequidad territorial^28^.

Destaca la infrautilización del SEM como recurso asistencial ante enfermedades graves y tiempo-dependientes como el SCA. Solo la mitad de la muestra usó esta vía, siendo altamente recomendado por su capacidad diagnóstica, asistencial y estar integrado en un sistema en red con hospitales con dotación para tratamiento de reperfusión 24/7. Además, acudir a urgencias o consultar con atención primaria se asocia con mayores retrasos prehospitalarios y comporta riesgos potenciales ante la evolución en curso de un SCA^4^, ^6^, ^20^, ^23^.

Es necesario fomentar el conocimiento poblacional sobre la enfermedad cardiovascular en la mujer y promover la activación del SEM ante sintomatología sugestiva de SCA, en vez de buscar otras vías de atención^6^, ^29^.

Según la literatura, el binomio género-salud y los roles de género impactan en la percepción de la salud, el autocuidado y el uso de recursos, pudiendo retrasar la búsqueda de atención médica^1^, ^7^, ^12^, ^25^. Nuestro estudio incorpora el análisis de los roles de género^12^, ^30^ y refleja cómo el ISG no se asoció con el TD, aunque descriptivamente las mujeres con alta carga de género consultaron de forma precoz con mayor frecuencia. Asimismo, el síndrome ansioso-depresivo puede actuar como factor de confusión, haciendo que interpreten el SCA como una crisis de ansiedad o un empeoramiento del estado anímico, siendo un determinante crítico en el pronóstico y acceso al sistema, según la Sociedad Europea de Cardiología^31^.

Priorizar el enfoque de género en políticas públicas, docencia, investigación y práctica clínica motivaría cambios a nivel conductual y social^25^, ^30^, ^32^. En este sentido, la atención primaria es el ámbito idóneo para liderar esta integración, permitiendo identificar de forma precoz sesgos de género y reorientar el autocuidado.

Como limitaciones destacan la naturaleza unicéntrica, el reducido tamaño muestral que ha podido limitar la evaluación de posibles asociaciones como la presencia de diabetes mellitus o de dolor epigástrico. Existe un posible sesgo de selección/supervivencia al excluir a las pacientes de mayor gravedad clínica. Asimismo, la dicotomización del TD por la mediana conlleva cierta pérdida de información y no constituye un punto de corte clínico, sino una medida relativa al comportamiento de la muestra. Sin embargo, se consideran fortalezas la recogida estructurada y consecutiva de datos, así como el análisis de factores desde diferentes perspectivas y dimensiones.

En conclusión, el retraso en la solicitud de asistencia sanitaria en mujeres con SCA se asocia con factores sociodemográficos, clínicos y, fundamentalmente, cognitivos. Es prioritario integrar estos hallazgos en el diseño de estrategias e intervenciones comunitarias para reducir el TD y mejorar la salud cardiovascular de la mujer.

**Lo conocido sobre el tema**

• La enfermedad cardiovascular causa el 35% de las muertes anuales en mujeres, siendo el síndrome coronario agudo la primera causa de mortalidad.
• Las mujeres se consideran un grupo de riesgo al presentar demoras prehospitalarias significativamente mayores que los hombres, favoreciendo presentaciones clínicas evolucionadas y peor pronóstico.
• Existe la necesidad de identificar qué factores se relacionan con el retraso en la solicitud de asistencia sanitaria en mujeres, desde un enfoque multidimensional que incorpore la perspectiva de género.

**Qué aporta este estudio**

• Introduce el índice de sobrecarga de género y revela que el rol de cuidadora principal y las cargas familiares se concentran significativamente en las mujeres menores de 70 años.
• Muestra que los principales factores asociados al retraso son la ausencia de percepción de gravedad durante el evento y residir fuera del núcleo urbano, observando una tendencia marginal hacia la demora en pacientes con síndrome ansioso-depresivo, mientras que presentar mareo-inestabilidad se relacionó con una consulta precoz.
• Subraya la necesidad de fomentar el conocimiento poblacional sobre la enfermedad cardiovascular en la mujer y propone a la atención primaria como el ámbito idóneo para aplicar el enfoque de género, identificar sesgos clínicos precozmente y optimizar los tiempos de respuesta mediante intervenciones comunitarias.

## Declaración de contribución de autoría CRediT

M.P.-V. se encargó de la conceptualización, curación de datos, análisis formal, investigación, metodología, administración del proyecto, supervisión, visualización y redacción del borrador original. S.P.-O. y N.R.-M. participaron en la conceptualización, curación de datos, investigación, administración del proyecto, visualización y redacción-revisión y edición. M.V. colaboró en la conceptualización, visualización y redacción-revisión y edición. R.A. y A.Z. contribuyeron en la conceptualización, metodología, validación, visualización y redacción-revisión y edición del manuscrito.

## Financiación

Este trabajo fue apoyado por el Hospital Clínic de Barcelona mediante la 5.ª edición de la Beca de Intensificación Investigadora para Enfermeras, concedida a la investigadora principal. La entidad financiadora no ha tenido participación alguna en el diseño del estudio, la recogida, el análisis o la interpretación de los datos, ni en la redacción del manuscrito o en la decisión de enviar el artículo para su publicación.

## Consideraciones éticas

El estudio fue aprobado por el Comité de Ética de la Investigación con Medicamentos del Hospital Clínic de Barcelona (código de registro: HCB/2021/1221) y se llevó a cabo de acuerdo con la Ley de Investigación Biomédica 14/2007, en el marco de la Declaración de Helsinki.

## Consentimiento informado

Toda la información clínica se trató de forma confidencial y anonimizada, y se obtuvo el consentimiento informado de las participantes antes de su inclusión en el estudio.

## Declaración sobre el uso de la IA generativa y de las tecnologías asistidas por la IA en el proceso de redacción

Durante la preparación de este trabajo las autoras no han utilizado herramientas de IA generativa.

## Conflicto de intereses

La investigadora principal declara haber recibido apoyo para la investigación mediante una beca de intensificación del Hospital Clínic de Barcelona (liberación de carga asistencial) y una ayuda al doctorado del Consejo General de Enfermería. El resto de las autoras declaran no tener conflicto de intereses.

## References

[1] Vogel B., Acevedo M., Appelman Y., Bairey Merz C.N., Chieffo A., Figtree G.A. (2021). The Lancet women and cardiovascular disease Commission: Reducing the global burden by 2030. Lancet.

[2] Chandrasekhar J., Gill A., Mehran R. (2018). Acute myocardial infarction in young women: Current perspectives. Int J Womens Health.

[3] Mirzaei S., Steffen A., Vuckovic K., Ryan C., Bronas U.G., Zegre-Hemsey J. (2020). The association between symptom onset characteristics and prehospital delay in women and men with acute coronary syndrome. Eur J Cardiovasc Nurs.

[4] Sederholm Lawesson S., Isaksson R., Ericsson M., Angerud K., Thylén I. (2018). Gender disparities in first medical contact and delay in ST-elevation myocardial infarction: A prospective multicentre Swedish survey study. BMJ Open.

[5] Davis L.L. (2017). A qualitative study of symptom experiences of women with acute coronary syndrome. J Cardiovasc Nurs.

[6] Byrne R.A., Rossello X., Coughlan J.J., Barbato E., Berry C., Chieffo A. (2023). 2023 ESC Guidelines for the management of acute coronary syndromes. Eur Heart J.

[7] Coll-Vinent B. (2022). Being a woman: A risk factor for poorer health outcomes. Emergencias.

[8] Giordano V., Guillari A., Sansone V., Catone M., Rea T. (2024). Women acute myocardial infarction-identifying and understanding the gender gap (WAMy-GAP): A study protocol. Healthcare (Basel).

[9] Miró O., Martínez-Nadal G., García A., López-Barbeito B., Carbó M., Placer A. (2022). Análisis individualizado por sexo de los factores asociados a la clasificación inicial errónea en urgencias del dolor torácico como no coronario. Emergencias.

[10] Bollen K., Lennox R. (1991). Conventional wisdom on measurement: A structural equation perspective. Psychol Bull.

[11] Giordano V., Mercuri C., Simeone S., Rea T., Virgolesi M., Nocerino R. (2025). Behavioral delays in seeking care among post-acute myocardial infarction women: A qualitative study following percutaneous coronary intervention. Front Glob Womens Health.

[12] Mies Vargas C. (2023). El género como determinante social de la salud y su impacto en el desarrollo sostenible. Universitas.

[13] Barros A.J., Hirakata V.N. (2003). Alternatives for logistic regression in cross-sectional studies: an empirical comparison of models that directly estimate the prevalence ratio. BMC Med Res Methodol.

[14] Von Elm E., Altman D.G., Egger M., Pocock S.J., Gøtzsche P.C., Vandenbroucke J.P. (2007). The Strengthening the Reporting of Observational Studies in Epidemiology (STROBE) statement: Guidelines for reporting observational studies. Lancet.

[15] Sugranyes G., Sebastià M.C., García-Delgar B., Forcadell E., Coll-Vinent B., Grupo de Trabajo de Género en Salud del Hospital Clínic de Barcelona (2023). Consideraciones respecto al uso de la variable sexo/género en investigación para avanzar hacia una buena praxis: Decálogo PROGÉNEROS. Emergencias.

[16] World Medical Association (2025). World Medical Association Declaration of Helsinki: Ethical Principles for Medical Research Involving Human Participants. JAMA.

[17] Killip T., Kimball J.T. (1967). Treatment of myocardial infarction in a coronary care unit. A two year experience with 250 patients. Am J Cardiol.

[18] Holtzman J.N., Kaur G., Hansen B., Bushana N., Gulati M. (2023). Sex differences in the management of atherosclerotic cardiovascular disease. Atherosclerosis.

[19] Mesas C.E., Rodrigues R.J., Mesas A.E., Feijó V.B.R., Paraiso L.M.C., Bragatto G.F.G.A. (2018). Symptoms awareness, emergency medical service utilization and hospital transfer delay in myocardial infarction. BMC Health Serv Res.

[20] Abellas Sequeiros M., García Sebastián C., Zamorano Gómez J.L. (2021). Complicaciones del infarto agudo de miocardio. Medicine.

[21] De la Torre-Hernández J.M., Aldama-López G., Andrea R., Corbí-Pascual M., Cordero A., Fernández Olmo M.R. (2025). Recomendaciones para la optimización de la atención a los pacientes tras infarto agudo de miocardio. Proyecto MIMIC. REC CardioClinics.

[22] Arrebola-Moreno M., Petrova D., García-Retamero R., Rivera-López R., Jordan-Martínez L., Arrebola J.P. (2020). Psychological and cognitive factors related to prehospital delay in acute coronary syndrome: A systematic review. Int J Nurs Stud.

[23] Gulati M., Levy P., Mukherjee D., Amsterdam E., Bhatt D.L., Birtcher K. (2021). 2021 AHA/ACC/ASE/CHEST/SAEM/SCCT/SCMR Guideline for the evaluation and diagnosis of chest pain: Executive summary. J Am Coll Cardiol.

[24] Sambola A., Soriano-Colomé T. (2022). Es el momento de cambiar en urgencias la evaluación del dolor torácico en la mujer. Emergencias.

[25] Fernández-Friera L., Pedreira Pérez M., Campuzano Ruiz R., Gámez J.M., Rodríguez Padial L., Ortiz Cortés C. (2025). Comité Científico del proyecto Mujer y Corazón de la Sociedad Española de Cardiología. Percepción y conocimiento de las ECV entre las mujeres en España: encuesta nacional sobre concienciación, riesgo y estilo de vida. Rev Esp Cardiol.

[26] Parellada-Vendrell M., Pérez-Ortega S., Romeu-Mirabete N., Prat-Masana M., Venturas M., Zabalegui A. (2025). Analysis of clinical and epidemiological profiles as predictors of complications in women admitted to the acute cardiac care unit for acute coronary syndrome. Womens Health Rep (New Rochelle).

[27] Manzo-Silberman S., Hawranek M., Banerjee S., Kaluzna-Oleksy M., Alasnag M., Paradies V. (2024). Call to action for acute myocardial infarction in women: international multi-disciplinary practical roadmap. Eur Heart J Open.

[28] Urbanos-Garrido R. (2016). La desigualdad en el acceso a las prestaciones sanitarias. Propuestas para lograr la equidad. Gac Sanit.

[29] De Diego O., Rueda F., Carrillo X., Oliveras T., Andrea R., El Ouaddi N. (2023). Performance analysis of a STEMI network: Prognostic impact of the type of first medical contact facility. Rev Esp Cardiol (Engl Ed).

[30] Velasco Arias S. (2009). https://www.sanidad.gob.es/organizacion/sns/planCalidadSNS/pdf/equidad/recomendVelasco2009.pdf.

[31] Bueno H., Deaton C., Farrero M., Forsyth F., Braunschweig F., Buccheri S., ESC Scientific Document Group (2025). 2025 ESC Clinical Consensus Statement on mental health and cardiovascular disease: Developed under the auspices of the ESC Clinical Practice Guidelines Committee. Eur Heart J.

[32] Hawkes S., Sy E.A., Barker G., Baum F.E., Buse K., Chang A.Y. (2025). Achieving gender justice for global health equity: The Lancet Commission on gender and global health. Lancet.

